# A mathematical approach to study and forecast racial groups interactions: deterministic modeling and scenario method

**DOI:** 10.1007/s11135-017-0581-9

**Published:** 2017-10-03

**Authors:** Goran Dominioni, Addolorata Marasco, Alessandro Romano

**Affiliations:** 1 Rotterdam Institute of Law and Economics, Erasmus University Rotterdam, Burgemeester Oudlaan 50, 3000 DR Rotterdam, The Netherlands; 2 Department of Mathematics and Applications, University of Naples Federico II, Via Cintia, 80126 Naples, Italy; 3 China University of Political Science and Law, Xitucheng Road 25, Haidian District, Beijing, China; 4 Yale Law School, 127 Wall St., New Haven, CT 06511 USA

**Keywords:** Racial interactions, Deterministic modeling, Scenario method, Logit model, Lotka–Volterra systems

## Abstract

Globalization and migratory fluxes are increasing the ethnic and racial diversity within many countries. Therefore, describing social dynamics requires models that are apt to capture multi-groups interactions. Building on the assumption of a relationship between multi-racial dynamics and *socioeconomic status (SES)*, we introduce an aggregate, contextual, and continuous index of SES accounting for measures of income, employment, expected life, and group numerosity. After, taking into account that groups’ SES assumes the form of a *logit model*, we propose a Lotka–Volterra system to study and forecast the interaction among racial groups. Last, we apply our methodology to describe the racial dynamics in the US society. In particular, we study the kind and the intensity of Asians–Blacks–Natives–Whites interactions in the US between 2002 and 2013. Moreover, we forecast the evolution of groups’ SES and how interracial relations will unfold between 2013 and 2018 and in three alternative stylized scenarios.

**Electronic supplementary material:**

The online version of this article (doi:10.1007/s11135-017-0581-9) contains supplementary material, which is available to authorized users.

## Introduction

Ethnic and racial diversity is rapidly increasing within many countries. The transformation of United States into a prismatic society constitutes a paradigmatic example of this phenomenon. And indeed, according to Census Bureau projections by 2042 non-Hispanics Whites will amount for less than 50% of the population (U.S. Census Bureau 2008). Prismatic societies pose a great challenge for social scientists, as inter-groups studies have often built on the two groups paradigm (Abascal 2015). Yet, the methodologies used to analyze the two-party case are not suited for triads (or multi-groups cases), as the latter “entails a *transformation*, not simply an *extension*, of the two-party case” (Abascal 2015). For this reason, developing quantitative models to understand, predict and control multi-groups interracial dynamics is a compelling priority. To take on this challenge, we propose a mathematical approach based on a deterministic model of the Lotka–Volterra (LV) type to study multi-group interactions from a dynamic perspective. This mathematical model, previously introduced in Marasco et al. (2016a, b), provides a methodology to study the kind (e.g. mutualism, competition, etc.) and the intensity of the interactions among groups and to forecast future dynamics in a scenario framework. We argue that the joint use of the proposed LV model and the scenario methodology offers a key to understand, interpret, and predict multi-groups dynamics.

In particular, we study the dynamical interactions among Asians, Blacks, Natives and Whites within the United States. The analysis is structured as follows. We start by proposing an aggregate index of socioeconomic status (SES) comprising measures of income, employment, expected life, and numerosity of each group for the period 2002–2013. Then, we show that these SES indices can assume the form of a *logit model*, and that therefore the corresponding *SES functions can be linked to the competitive roles in the LV dynamic framework* as shown in Marasco et al. (2016a). Indeed, in our approach the SES functions allow us to highlight and quantitatively measure the interactions among racial groups. Therefore, using the proposed model to study the dynamics of the SES indices allows us determine the kind and the intensity of the interactions (in terms of changes in the respective SES) among Asians, Blacks, Natives and Whites during the time interval considered. Last, we forecast the evolution of groups’ SES and how interracial relations will unfold in three stylized scenarios. In the first scenario we study what is the magnitude of the policies that would be necessary to prevent Whites from losing SES shares. In the second scenario we perform a similar analysis, but the goal is to ensure that Blacks’ SES shares become at least equal to that of Asians. In the third scenario we simulate a period of economic expansion. We disregard the normative appeal of each scenario, as our intention is merely to offer a quantitative measure of the changes required to achieve a given goal or to investigate the impact on SES’ dynamics of a change in some components of the SES.

The proposed model is general and applies for any number of racial groups and for any continuous measure of SES. Therefore, while in this article we focus on Asians, Blacks, Natives and Whites for reasons of data comparability (e.g. the classification of Latinos changes across data sources), the analysis can be replicated including other racial/ethnic groups. Similarly, it is possible to adopt a different index for groups’ SES.

We remark that, although formally the SES functions play the role of the utility functions in a logit model, our methodology in no way implies that the individuals belonging to a racial group consciously organize to maximize the utility of the group to which they belong. Instead, the analysis only requires three milder conditions to hold. First, despite being indistinguishable from an anthropological point of view (AAA 1998), racial groups must be a meaningful unit of analysis from a sociological perspective and in the public policy debate. This is confirmed by the countless domains that are relevant to define the SES of an individual in which there are persistent and significant differences among races (Waters and Eschbach 1995; Smedley and Smedley 2005) [e.g. life expectancy (Day 1996) and unemployment (U.S. Bureau of Labor Statistics 2012)]. Second, given that SES is inherently a relative concept (i.e. the SES of an individual can only be interpreted with respect to the SES of the other individuals) (Brown 2007), we claim that variations in the SES of one group have an influence on the SES of other groups. For instance, an individual with a given level of income can be considered upper-class in contexts in which the average income is significantly lower, whereas the same level of income could qualify her as low-class in contexts in which the average income is higher. Therefore, if the income of all racial groups but one significantly increases over time, the socioeconomic status of the group that is left behind will worsen even though its income has not decreased (e.g. average prices will be higher). Similarly, despite the fact that the life expectancy of Blacks in United States has increased over the last decades, the literature considers that Blacks suffer from poor health conditions because they live less (and in worse health conditions) than Asians (Hayward and Heron 1999). The fact that Blacks have a lower life expectancy than other groups sharing the same environment is an indicator that their socioeconomic status is relatively lower in this dimension. The same logic applies to group size. The political influence of a racial group is affected by its relative numerosity, if anything due to the share of votes that it can cast at the elections. For instance, in a country with a small population a racial group composed by a few thousands of individuals might have a relevant voice. The same group would have a negligible influence in a country with a large population. In this vein, if the size of one group increases it worsens the relative position of the other groups. Third, we argue that the socioeconomic status is a determinant of the kind of interaction among groups. This is corroborated by research showing that racial attitudes shape (Huffman and Cohen 2004) and are shaped (Taylor and Reyes 2014) by the SES of the racial groups. In other words, we build on the assumption that there is a relationship between multi-racial dynamics and socioeconomic status.

The main findings of the paper are the following. First, we find that the LV model introduced in Marasco et al. (2016a) can accurately describe and forecast groups interactions provided that the SES index for each group are rewritten as a logit model. Indeed the measures of error considered indicate that the model is “highly accurate”. Second, the kind of interaction among the racial groups changes over time, indicating that studies that do not use panel data can only take snapshots of reality. Third, our approach confirms that the interactions among racial groups are influenced by macroeconomic factors. In particular, we find that during the 2008 financial crisis all racial groups engage in rivalrous interactions and that the intensity of competition is modulated by the severity of the crisis. Fourth, we find that only very drastic interventions can prevent the SES share of Whites from declining or allow Blacks to enjoy the same SES share of Asians. Last, an economic boom only marginally affects SES shares, while increasing the level of mutualism within the society. The structure of the paper is the following: Sect. 2 covers some preliminary methodological issue and the background literature. Section 3 illustrates the data and the method. The results are presented in Sect. 4. Section 5 concludes and discusses potential extensions of this research.

## Preliminary methodological considerations and background literature

### Background literature

At a general level, this work relates to the literature that analyzes racial groups SES dynamics. In an influential article Hirschman studies the evolution of the SES of Asians, Blacks, Hispanics and White men in the United States between 1960 and 1976 (Hirschman and Wong 1984). His main finding is that the socioeconomic status of high SES minorities tends to converge, or even surpass, that of the Whites, whereas disadvantaged minorities (e.g. Black) constantly lag behind. Although many decades have passed, we find that very similar dynamics still characterize American society. Other studies concentrate on the evolution of one of the dimensions of SES over time (generally wages) finding that differences across racial lines tend to be stable over time [see Leicht (2008) and references therein]. We contribute to this literature by studying from a dynamic perspective the interactions among the SES of the various groups.

Two other strands of literature that are relevant to our analysis are studies investigating (i) how the SES of one group influences the attitude of out-group members toward that group and (ii) how the attitudes of the out-group members toward a group influence the SES of that group. Taken together, (i) and (ii) show that the interactions among groups are influenced by their respective SES via attitudes. However, while there is a general consensus in the literature that racial group SES affects racial interactions (Branton and Jones 2005; Taylor and Reyes 2014), scholars disagree on the direction of this effect. On the one hand, literature on racial group competition and threat argues that minorities with high SES foster negative attitudes in the dominant White group (Blumer 1958; Blalock 1967). This occurs because as minorities improve their social standing in society Whites perceive that they are encroaching their privileged position. Conversely, the group contact hypothesis predicts that higher SES levels of racial minorities can trigger positive attitudes in the majority group (Allport 1954). In this view, an equal social standing facilitates positive inter-group contacts. Looking at the SES-attitudes relationship from the opposite perspective, various strands of research investigate impact of attitudes on the allocation of resources among racial groups (Pager and Karafin 2009; DeSante 2013). This literature shows that racial attitudes create differences in key determinants of SES across racial groups [e.g. job positions (Pager and Karafin 2009), wages (Holzer and Ihlanfeldt 1998; Huffman and Cohen 2004), and government assistance (DeSante 2013)]. To summarize, variations in the SES of a racial group can affect the distribution of resources across racial groups. This, in turn, can trigger shifts in SES measures of the various groups within a society.

Another related strand of literature investigates directly how variations in one component of the SES of a group affects the SES of other groups (Cohen 1998). In this regard, it is important to distinguish two ways in which these effects can take place. On the one hand, changes in the socioeconomic standing of a group always have an effect on the SES of the other groups because SES is an inherently relative concept (Brown 2007). If one group improves its condition while the other remains stable, the position of the latter becomes relatively worse. On the other hand, research on the interactions between racial groups’ SES highlights that variations in SES measures of a racial group can trigger variations in SES measures of other racial groups in absolute terms. For instance, variations in the size of minority populations may lead to reductions/increases in the economic well-being of different racial groups (Tigges and Tootle 1993; Cohen 1998; Albrecht et al. 2005). In this regard, Cohen (1998) finds a negative relation between concentrations of Blacks and their earnings. Moreover, he finds a positive relation between the number of Blacks and the earnings of Whites. Therefore, there is a relationship between groups’ SES that goes beyond the mere shift in the relative position of the groups.

On this background, the emergence of a prismatic society complicates the analysis, because a multi-racial society is a fertile environment for the emergence of nuanced relationships among racial groups. And indeed, a growing body of literature is investigating social patterns in a multi-groups framework (Wilkinson 2014). The present work contributes to this literature by offering a rigorous mathematical approach to test in a quantitative and replicable way how *n* racial groups interact and influence each other SES.

### Conceptual framework of the analysis

An important methodological premise to this study is defining in a clear and detailed way the SES measure, because SES is a multidimensional and contextual concept that can be measured using many different indicators (Braveman 2005; Berkowitz 2015; Berzofsky et al. 2015). In particular, traditional measures of SES include education, income, employment—sometimes called the “big three”—but also wealth, household tenure, parental education, and so on. SES-related measures are sometimes used as single items or suitably combined (Branton and Jones 2005; Braveman 2005; Berkowitz 2015; Berzofsky et al. 2015), and are often operationalized as a single ordinal categorical variable (e.g., poor/nonpoor, less than high school/high school/more than highschool, or low/middle/upper wealth). Moreover, measures of SES can have a different scope and range from a neighborhood (identified via ZIP codes, census tracts, and census blocks) to areas as large as states and regions. As both single and composite measures of SES have some disadvantages, it is widely understood that there cannot be one universally accepted indicator, and the debate is still open on which are the most reliable (Alsabbagh 2016). It was noted that (i) the indicators should carry as much relevant information as possible, (ii) the indicators used should be clearly spelled out, including their limitations, (iii) and the impact of non accounted factors should be explicitly stated (Braveman 2005). We try to follow these guidelines to build our measure of SES. In detail, we adopt a multi-dimensions approach that accounts for measures of income, employment, group numerosity, and life expectancy. We focus on income and employment because they constitute natural dimensions to study interracial dynamics and the reciprocal impact of the various groups on their respective SES. Moreover, recent studies show that measures of income, even considered alone, have a high predictive power on relevant outcome variables (Alsabbagh 2016).

In addition, to develop a more comprehensive indicator of SES, we aggregate also information relative to groups’ political power and health status. We account for the former by considering group numerosity, because size can positively influence the political strength of a group in a democratic society. This is even more so in societies, such as the American one, in which minorities’ political turnout is positively correlated with increases in their relative group size (Fraga 2016). We account for the latter by including information on life expectancy, which is widely recognized as an indicator of various dimensions of SES [e.g. exposure to pollutants, eating habits, access to health services, exposure to violent crimes, etc. (Harper 2007; Lynch and Kaplan 2000)]. Admittedly, our indicator overlooks some important component of SES, like education and wealth. We do not include these factors due to data availability and because they cannot be aggregated in a straightforward way with the other measures of SES considered. For example, unlike the variables that we consider, education is not a continuous variable. And indeed, the “amount” of education is traditionally expressed using categories (e.g. high school, bachelor, master etc.). Then, in order to include a measure of education in the proposed SES index it would be necessary to build an ad hoc continuous version of the “amount” of education (e.g. as a weight varying in the interval [0,1]).

However, we remark that in principle our approach is well suited also when accounting for measures of wealth and education. Similarly, provided that the relevant data is available, the analysis can be replicated at a State level or even at a county level. While the indicators chosen are far from foreign to the literature, we adopt a different strategy to aggregate them. Instead of obtaining the composite SES measure as a weighted average of their single indicators (Sackett 2009; Higdem 2016), we opt for a multiplicative form. This choice allows us to approximate the behavior of American courts in tort cases when calculating damages awards. As noted by Avraham and Yuracko, race-based tables of wage and life expectancy are commonly used to define the compensation owed to a victim of a tort (Avraham and Yuracko 2017). Assume that in a car accident a White and a Black child of the same age die. Simplifying to the extreme, if these tables are used a court will determine compensation multiplying the predicted wage of each child for his expected work-life (adjusted according to the specific circumstances of the case). Because Whites generally live longer and have higher wages, the parents of the White child will be awarded higher damages. This approach has a relevant practical impact on how resources are allocated within the society. For instance, *ceteris paribus* for a firm it is cheaper to pollute in a “Black neighborhood” than in a “White neighborhood” because the expected liability is lower (Avraham and Yuracko 2017). In turn, the choice of the firm to locate in a Black neighborhood further reduces the SES of the Blacks, as an increased pollution is likely to decrease life expectancy. Due to the practical relevance of these considerations, we build our SES indicator following the same logic and multiply income, expected life and employment rate. Last, we correct the result of this product for the size of the group.

As standard manipulations allow us to write each SES index as a logit model, we perform the quantitative analysis using the class of LV models introduced in Marasco et al. (2016a). These models are a powerful tool to study and forecast the interaction among different entities in a given environment. At a general level, LV models have the advantage of being able to capture every possible kind of interaction (i.e. mutualism, predator-prey, commensalism, competition, amensalism, and neutralism). For this reason, they are widely used in natural sciences, and are becoming more and more widespread in a variety of domains in social sciences [see Modis (1999), Romano (2013), Marasco et al. (2016a) and references therein]. There are two main factors that explain why traditionally LV models have been used more frequently in natural sciences. First, the most used LV models are *autonomous*, i.e., the model equations contain only constant coefficients as in Tsai and Li (2009), Chiang (2012), Lin (2013), Lakka (2013), Duan et al. (2014), Cerqueti et al. (2015), thus, the kind and the intensity of the interaction is assumed to be constant over time. Intuitively, this is a greater limitation for studies in social sciences than for *some* studies in natural sciences. For example, it is very unlikely that sheeps and wolves change the way in which they interact, and therefore modeling them respectively as preys and predators for the whole time horizon is a perfectly reasonable choice. On the contrary, phenomena in social sciences are usually characterized by a high variability of competitive roles. For instance, an appropriate marketing campaign can turn a firm that used to be a prey of another into a predator. Similarly, we expect that ethnic groups do not have a constant pattern of interaction (Brandt et al. 2014), thus autonomous LV systems are generally not an appropriate choice to model this social dynamics. Second, in most cases the analytical solutions of LV models—especially in the nonautonomous case—are not known in a closed form. Therefore, the parameters of the model have to be estimated using expensive numerical fitting procedures. This is not always possible in many domains of social sciences that are plagued by data scarcity. The LV model presented in Marasco et al. (2016a), Romano (2016) overcomes both problems. On the one hand, it is a nonautonomous model and therefore the competing entities are allowed to change their competitive roles over time. On the other hand, the analytic solutions are known and therefore the quantitative analysis is significantly easier and requires less data. Importantly, the analytical solutions of this class of LV models are in the form of a *logit model* introduced by McFadden (1973), extensively used in every area of social sciences, and also in studies analysing socioeconomic status and race (Bayer and McMillan 2005). Therefore, besides its properties, this model has the additional advantage of being coherent with the mainstream approach to the quantitative study of many social phenomena. Differently from Marasco et al. (2016a), in this paper we can easily design the scenarios since the SES functions are linked in a known way to the components of groups’ SES (i.e., income, employment, group numerosity, and life expectancy).

### Scenario method in public policy

The scenario method consists in developing *“a set of hypothetical events set in the future constructed to clarify a possible chain of causal events as well as their decision points”* (Kahn and Wiener 1967). In particular, scenario planning allows to forecast future dynamics by presenting the crucial elements of a given problem in a systematic and coherent way (Burt and Heijden 2003; Amer et al. 2013). If appropriately applied, it is a powerful tool to approach complex problems characterized by a high degree of uncertainty in a more rational and effective way (Kahn 1962). Moreover, in settings dominated by uncertainty, scenarios are useful for *“highlighting implications of possible future system discontinuities, identifying nature and timing of these implications, and projecting consequences of a particular choice or policy decision”* (Amer et al. 2013). Not surprisingly, the literature identifies a correlation between the degree of uncertainty characterizing a given domain and the use of the scenario method (Malaska 1984). As in many policy domains the debate is dominated by the concept of uncertainty (e.g. environmental law (Sachs 2011), health law (Sadeleer 2006), financial law (Pacces and Romano 2015)), scenario planning can be a useful tool also for policy makers. We contribute to the scenario literature in two ways. First, many of the scenario applications in the public policy area often remain at a qualitative level. We complement this literature by proposing a deterministic modeling approach that translates into deterministic predictions the set of possible narratives. Second, we apply the scenario methodology to a domain that is extremely appropriate for scenario analysis due to its complexity and uncertainty, yet in which scenarios—to the best of our knowledge—have not been applied before.

## Materials and methods

### Data

We collect data on life expectancy, average income, employment rate, and total number of residents for four racial groups ($R_i,\,i=0,...,3$): Natives ($i=0$), Asians ($i=1$), Blacks ($i=2$) and Whites ($i=3$). In this data set, the racial group of Asian refer both to Asian and Pacific Islanders, Natives include American Indians, Eskimo and Aleut, and all racial groups refer both to Hispanics and non-Hispanics. In particular, we obtain data on life expectancy and numerosity from the U.S. Bureau of the Census (Day 1996).

The *life expectancy* $L_i{(t)}$ for the $i-$th racial group $R_i$, at time *t*, is obtained as follows

1 $$\begin{aligned} L_{i}{(t)}=\frac{N_i^{f}{(t)}\,L_i^{f}{(t)}+N_i^{m}{(t)}\,L_i^{m}{(t)}}{N_i^{f}{(t)}+N_i^{m}{(t)}}, \end{aligned}$$

where $N_i^{f}{(t)},N_i^{m}{(t)}$ represent the total number of female and male resident, respectively; whereas $L_i^{f}{(t)},L_i^{m}{(t)}$ are the corresponding mean life expectancies. We denote by $N_i{(t)}$ the total number of residents of the $i-$th racial group $R_i$, whereas $N{(t)}={\displaystyle \sum \limits _{i=0}^{3}} N_{i}{(t)}$ is the total number of residents.

To fill gaps in the dataset containing the annual values of $N_i^{f}{(t)},L_i^{f}{(t)},N_i^{m}{(t)}$, and $L_i^{m}{(t)}$, we construct an approximate function that interpolates the available data for each variable in Eq. (1) (years 2002–2005, 2010, 2015), then we use this function to find the missing values (years 2006–2009, 2011–2014). In particular, the fitting procedures are performed by using the following *Fourier series of order* *n*

2 $$\begin{aligned} a_{0}+\displaystyle \sum \limits _{r=1}^{n}\left( a_{r}\cos \frac{ r\pi t}{\tau }+b_{r}\sin \frac{r\pi t}{\tau }\right) \end{aligned}$$

where $$\tau =(2015-2002)+1$$, and $$n=2$$.

**Fig. 1:**
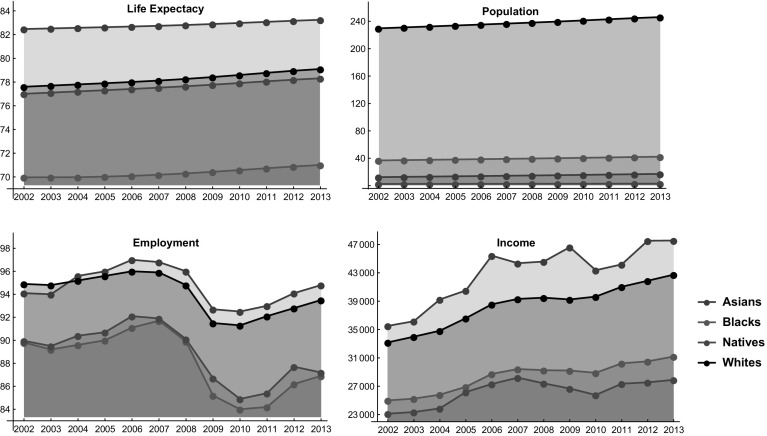
Life expectancy (age), total number of residents (millions), employment rate (%), and average income ($) for each racial group over the period 2002–2013

The data on *average income* $I_i$ per racial group for Asians, Blacks and Whites is taken from the website of the U.S. Census Bureau, https://www.census.gov/hhes/www/income/data/historical/people/.^1^ In particular, we take the Asians Alone, Blacks Alone and Whites Alone data at current dollar value. Last, the data on *employment* $E_i$ per racial group for Asians, Blacks and Whites is obtained from a report of the U.S. Bureau of Labor Statistics (2012). All the collected data are reported in Fig. 1.

### SES index, and logit model

Supposing that the ***SES index*** $S_{i}{(t)}$, at any time *t*, for each groups is a function of the variables $L_{i}{(t)}, E_{i}{(t)}, I_{i}{(t)},N_{i}{(t)}$, we propose the following analytical form

3 $$\begin{aligned} S_{i}{(t)}=\displaystyle \frac{L_{i}{(t)}\times E_{i}{(t)}\times I_{i}{(t)}\times \ln \left( 1+{100}N_{i}{(t)}/N{(t)}\right) }{M{(t)}},\quad i=0,\ldots ,3 \end{aligned}$$

where

$$\begin{aligned} M{(t)}={\sum \limits _{i=0}^{3}}\left[ L_{i}{(t)}\times E_{i}{(t)}\times I_{i}{(t)}\times \ln \left( 1+{100}N_{i}{(t)}/N{(t)}\right) \right] . \end{aligned}$$

We note that the function $\ln \left( 1+{100}N_{i}{(t)}/N{(t)}\right)$ is a weight function to account for the relative size of $i-$th racial group (see Fig. 14 in the “Appendix”).

**Fig. 2:**
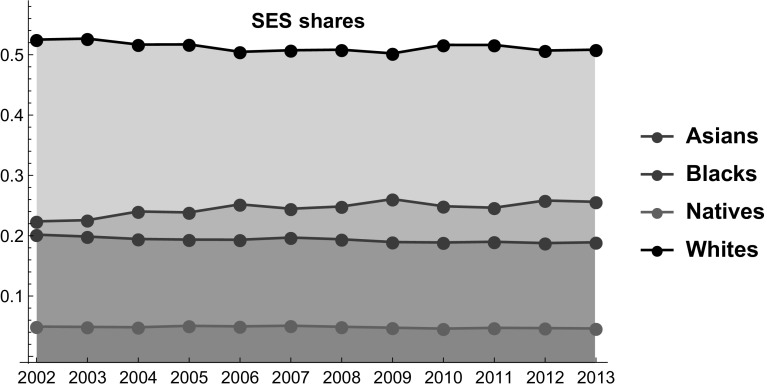
SES indices for each racial group over the period 2002–2013

To highlight the role of each variable in the aggregate indices $S_i{(t)}$, we summarize in Table 1 all data for the four racial groups in 2002. The SES indices over the period 2002–2013 are reported in Fig. 2 (see also Table 6 in the “Appendix”).

**Table 1:** Driving data and the SES index for each racial group in 2002

Racial group	Life expectacy (age)	Population (millions)	Employment (%)	Income ($)	SES (%)
Asians	82.4719	12.023	94.1	35,518	0.223783
Blacks	69.9651	36.366	89.8	25,002	0.201804
Natives	77.0088	2.469	89.95	23,128	0.0493768
Whites	77.6033	228.332	94.9	33,185	0.525036

In order to apply the LV model proposed in Marasco et al. (2016a) to the study of racial groups interactions, we need to write each SES index in the form of a *logit model*. As we see in Fig. 2, the role of the “outside good” is played by the racial group of “Natives” ($i=0$), because of their very low SES share. Then, introducing the positive functions

4 $$\begin{aligned} A_{i}{(t)}=\left[ L_{i}{(t)}\times E_{i}{(t)}\times I_{i}{(t)}\times \ln \left( 1+{100}N_{i}{(t)}/N{(t)}\right) \right] ,\quad i=0,\ldots ,3 \end{aligned}$$

standard manipulations allow to write Eq. (3) in the following form

5 $$\begin{aligned} S_{0}{(t)}=\frac{1}{1+{ {\displaystyle \sum \limits _{j=1}^{3}} }\exp (f_{j}(t) )},\quad S_{i}{(t)}=\frac{\exp (f_{i}(t) )}{1+{ {\displaystyle \sum \limits _{j=1}^{3}} }\exp (f_{j}(t) )},\quad i=1,2,3, \end{aligned}$$

where the *SES functions* $f_{i}$ write

6 $$\begin{aligned} f_{i}(t) =\ln \dfrac{A_{i}{(t)}}{A_{0}{(t)}},\quad i=0,...,3. \end{aligned}$$

We remark that, as in the *logit models*, the SES of the $i-$th racial group increases when its SES function $f_{i}(t)$ increases and decreases when the SES function $f_{j}(t)$ of any other group increases. The first relation captures the fact that the SES of a group is positively influenced by increases in the resources (e.g. health, jobs, income) available to the members of the group. The second relation derives from the inherently relative nature of SES. Since the status of one group can be determined only in relation to the one of the other groups, positive variations in the SES of one group necessarily negatively affect the SES of the other groups and vice-versa.

Moreover, due to Eqs. (4)–(6), each SES function $f_{i}(t)$ increases when one or more functions $L_{i}{(t)}, E_{i}{(t)}, I_{i}{(t)},N_{i}{(t)}$ increase and decreases when one or more functions $L_{j}{(t)}, E_{j}{(t)}, I_{j}{(t)},N_{j}{(t)}$ of any other group increase ($j\ne i$). How SES shares vary in response to variations of one or more SES functions can only be evaluated by numerical simulations (see Sect. 4.4).

### The Lotka–Volterra model

In the following we describe in detail the proposed LV model in which the Native play the role of the “outside good” (see Fig. 2). By assigning the Natives the role of the outside good we implicitly assume that their strength is too limited to significantly alter social dynamics at a macro-level.

If we assume that all the SES functions $f_{i}(t) ,i=1,...,3,$ are of class $C^{2}\left( \left[ t_{0},+\infty \right) \right)$ it is easy to demonstrate that Eq. (5) are the unique (global) solution of the following Cauchy problem

7 $$\begin{aligned} \left\{ \begin{array}{l} {\dot{S}}_{i}(t)=g_{i}(t) S_{i}(t)\left[ 1-S_{i}(t)\right] -\sum \limits _{j=1,j\ne i}^{3}g_{j}(t) S_{j}(t)S_{i}(t),\quad i=1,\ldots ,3, \\ S_{i}(t_{0})=\dfrac{\exp (f_{i}\left( t_{0}\right) )}{1+\sum \limits _{j=1}^{3}\exp (f_{j}\left( t_{0}\right) )} \end{array} \right. t\in \left[ t_{0},+\infty \right) \end{aligned}$$

where the dot denotes the derivative respect to time, $S_{i}(t)\ge 0$ represents the SES share of the $i-$th racial group at time *t*, $g_{i}(t)={\dot{f}}_{i}(t)$, and $S_{0}(t)=1-\sum \limits _{i=1}^{3}S_{i}(t)$. Eq. (7) describes the interaction between the $i-$th and $j-$th racial groups by means of their SES indices in a framework of competitive roles varying over time. The evolution of the SES share $S_{i}(t)$ of the $i-$th racial group is mathematically determined by two factors: the logistic growth rate function $g_{i}(t)$ and the competition functions $g_{j}(t)$ between the $i-$th and $j-$th racial groups. Last, according to Eq. (5), the maximum capacity related to the saturation value of each $S_{i}(t)$ is 1. We remark that, at any time, the competitive roles are determined by the signs of the functions $g_{i}(t)$. Hence, according to Table 2 the LV model (7) is able to capture all the possible kinds of competitive interactions. Then, owing to Eq. (6), the competitive roles between the $i-$th and $j-$th racial groups are determined by the signs of the functions

8 $$\begin{aligned} g_{i}(t) ={\dot{f}}_{i}(t)=\dfrac{d}{dt}\left( \ln \dfrac{A_{i}{(t)} }{A_{0}{(t)}}\right) =\frac{{\dot{A}}_{i}(t)}{A_{i}(t)}-\frac{{\dot{A}}_{0}(t)}{A_{0}(t)},\quad i=1,2,3, \end{aligned}$$

where the functions $A_i (t)$ are defined in Eq. (4).

**Table 2:** The competitive roles between any pair of competitors $S_{i}(t)$ and $S_{j}(t)$

$g_{i}$	$g_{j}$	Type of interation	Explanation
$+$	$+$	Pure competition	The competitors suffer from each other’s existence
−	$+$	Predator-prey	Predator benefits from preys, whereas preys suffer from predators
−	−	Mutualism	Symbiosis or a win–win situation
−	0	Commensalism	One benefits from the existence of the other, while the other remains unaffected
$+$	0	Amensalism	One suffers from the existence of the other, which is impervious to what is happening
0	0	Neutralism	No interaction

To clarify the meaning of the possible forms of interactions, let us focus on the two minorities considered: Asians and Blacks. If these groups stand in a relationship of mutualism an increase in the SES (share) of one group favors the growth of the other group SES (share). Importantly, even in presence of mutualism there is no guarantee that when Asians gain more SES shares also the SES share of the Blacks grows, as the beneficial effect of the growth of Asians’ SES on Blacks’ SES must be balanced with other factors. An example of mutualism emerging among minorities is the following case. There is empirical evidence that some Asian minorities are discriminated on the work place and that this discrimination also negatively affects their health (Castro et al. 2008). Similar findings have been reported for Blacks (Krieger and Sidney 1996). In our framework these forms of discrimination are bound to affect the SES of both groups, because measures related to employment and to health are considered in our SES indicator. Let us assume that one group becomes more influential in the political arena (for example due to an increased number of in-group members) and some of its representatives persuade the government to pass more effective anti-discrimination rules. In this case, also the other group will benefit and hence mutualism between the groups could exist. Competition is the other side of the coin: when one group gains the other loses. For instance, the access to health services in United States is a scarce resource and is largely tied to the economic status. If Asians improve their economic status they will be able to purchase better insurances and “consume” a larger share of the pie destined to health care services. This could negatively affect the Blacks that might experience a further decrease in the share of health care expenditures allocated to them. There is amensalism between Asians and Blacks if an increase in the SES of the Asians (resp. Blacks) negatively affect the SES of the Blacks (resp. Asians), whereas the SES of the Asians (resp. Blacks) is unaffected by variations in the SES of the Blacks (resp. Asians). Instead, there is commensalism between Asians and Blacks if an increase in the SES of the Asians (resp. Blacks) positively affect the SES of the Blacks (resp. Asians), whereas the SES of the Asians (resp. Blacks) is unaffected by variations in the SES of the Blacks (resp. Asians). It seems reasonable to assume that these interactions emerge when one of the two groups is extremely small, and hence it only has a negligible influence on the other. As the number of Asians, Blacks and Whites is significant, we do not expect to observe these forms of interactions. Predator-prey is a more nuanced interaction. In particular, it presupposes that an increase in the SES of the predator harms the prey, whereas an increase in the SES of the prey benefits the predator. Last, neutralism simply means that the SES of the two groups are completely independent.

**Fig. 3:**
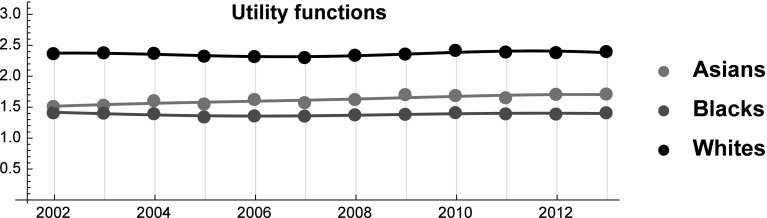
SES functions over the period 2002–2013

Equation (6) allows us to determine a discrete set of values for each SES function starting from historical data on SES shares (see Fig. 3). Therefore, the indirect determination of the analytical form of these functions is obtained by a fitting procedure using the Fourier series of order $n=2$ with $\tau =(2013-2002)+1$ [see Eq. (2) and Fig. 3].

#### Performance of the LV model on the historical data

**Fig. 4:**
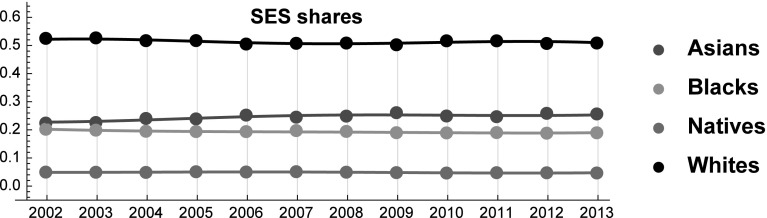
Estimated and observed SES shares over the period 2002–2013

To evaluate the fitting and forecasting performance of the proposed model, we use the *mean square error (MSE)*, and the *mean absolute percentage error (MAPE)*

$$\begin{aligned} MSE=\frac{1}{n}\sum \limits _{i=1}^{n}\left( h_{i}-p_{i}\right) ^{2},\quad MAPE=\frac{1}{n}\sum \limits _{i=1}^{n}\left| \frac{h_{i}-p_{i}}{h_{i}} \right| 100\%, \end{aligned}$$

where $h_{i}$ and $p_{i}$ are the historical and predicted values, respectively (Fig. 4).

**Table 3:** MSE, and MAPE for our model over the period 2002–2013

Racial group	Asians	Blacks	Natives	Whites
MSE	$2.504\times {10}^{-5}$	$2.617\times {10}^{-6}$	$5.172\times {10}^{-7}$	$1.548\times {10}^{-5}$
MAPE	1.925	0.604	1.376	0.682

The values of the MSE and the MAPE confirm that the model is “highly accurate” in describing interracial dynamics (Table 3). We recall that the forecasting accuracy by mean of MAPE can be classified into four levels: $<10\%$ (highly accurate), $10{-}20\%$ (good), $20{-}50\%$ (reasonable), and $>50\%$ (inaccurate) (Lewis 1982; Makridakis et al. 1979, 1984).

We note that LV models (autonomous and nonautonomous) have gained in popularity in time-series forecasting due to their simplicity and ability to characterize a real system by using few data points (e.g. Chiang 2012; Lin 2013). Nevertheless, we examine some standard forecasting methods, such as those belonging to ARIMA, GARCH, and SARIMA families, and compare them with our LV model to highlight the forecasting performances under the constraint of few data (not shown). Indeed, due to the paucity of data, we focus on ARIMA models for the dynamics of Asians and Blacks and compare them with LV models using the Mann–Whitney tests at significance level of 0.05.^2^

**Fig. 5:**
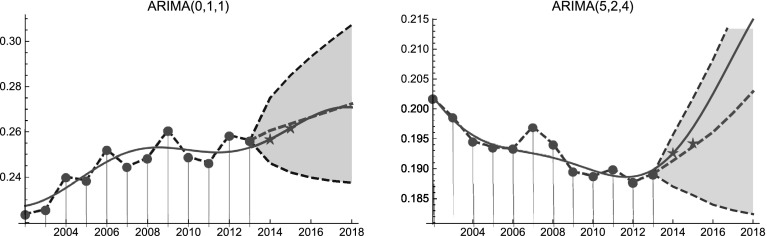
SES shares of Asians (left panel) and Blacks (right panel). Continuous and dashed lines refer to LV and ARIMA models, respectively. Star symbols represent the observed SES shares in the years 2014 and 2015. Blue and red regions represent 95% prediction bands. (Color figure online)

According to Akaike (AIC), Bayesian (BIC), and Schwartz–Bayes (SBC) information criteria, the SES shares of Asians are well described by an ARIMA process of parameters $p=0,d=1,q=1$ (AIC value $=-111.882,$ BIC value $=-109.84,$ SBC value $=-110.427$). The forecasted data obtained from LV and ARIMA models are statistically indistinguishable (*p* value of the Mann–Whitney test is 0.83). As we see in Fig. 5 (left panel), both models are consistent with the observed SES shares of Asians in the years 2014 and 2015. Similarly, choosing for the SES shares of Blacks the ARIMA(5, 2, 4) process (AIC value $=-124.794$ BIC value $=-131.551,$ SBC value $=-119.46$) we obtain the behavior in Fig. 5 (right panel). Although the SES shares of Blacks obtained by LV model belong to the upper prediction band, also in this case the forecasted data obtained from LV and ARIMA models are statistically indistinguishable (*p* value of the Mann–Whitney test is 0.83).

We remark that differently from forecasting statistical processes, the proposed LV methodology provides information about competitive roles (i.e. it highlights the kind and intensity of the interactions among populations) both in the past and in the future. Moreover, the procedures required in a statistical approach to design future scenarios (see Sects. 3.4, 4.2, 4.3, 4.4) would be extremely complex. This is especially true with regards to the possibility of controlling the considered phenomenon (see Scenarios SW1 and SB1) and evaluating the effect of any (quantitative) variation in one or more of the elements composing the SES (see Sect. 4.4).

### Scenario methodology and development

Although there are different approaches, it is frequent to divide the scenario analysis into five main steps (Foster 1993; Dong et al. 2013). Following this structure, we begin our analysis by identifying the major driving forces and the key variables that are likely to influence how groups interact and their SES (see Table 4). We note that the most part of these variables depend on each other.

**Table 4:** Main driving forces and variables to study racial groups interactions

Main driving forces	Variables
Demography	*life expectancy, numerosity*, immigration, age, distribution on the territory,...
Economy	relative and absolute wealth, *income*, *employment rate*, international context, knowledge based economy,...
Political/legislative	affirmative action policies, education policies, housing policies, health reforms, fiscal policies, criminal system reform,...
Culture	education, media, schools programs, political discourse,...
Environmental	climate change, pollution levels,...

Each of these driving forces deserves special attention to understand how inter-groups dynamics will unfold. For instance, affirmative action policies have been shown to effectively benefit minorities by fostering their employment (Miller and Segal 2012). In addition, affirmative action increases diversity in the workplace (Kalev et al. 2006), thus facilitating interracial contact, which is an important determinant of interracial attitudes. Similarly, media play a key role in shaping racial attitudes and stereotypes (Weisbuch et al. 2009). However, for reasons of tractability and data availability we limit our focus to four of these variables. In particular, we concentrate on measures of income, employment rate, life expectancy and group numerosity. The second step of the scenario analysis consists in developing the scenario logic and the respective story lines. During the first two steps the analysis is prevalently qualitative and it is generally conducted via interviews, surveys and workshops. The third step consists in quantifying the future developments of the main driving forces and the corresponding variables. For instance, a “low numerosity” among Whites could be quantified in Whites representing only $60\%$ of the population in the near future. Alternatively, it can be studied a “high numerosity” scenario in which the share of the Whites is above current levels. These measures are then used in the fourth step to perform a quantitative analysis of the main variables of interest. Last, in the fifth step the scenarios are updated and refined on the basis of the availability of new data or knowledge.

The LV model presented in this work and introduced in Marasco et al. (2016a) is suited to perform the last three steps of the scenario analysis, and especially the fourth one. In fact, during the fourth phase quantitative methods have a comparative advantage over qualitative ones in providing accurate forecasts. Moreover, we improve on Marasco et al. (2016a) because we identify the SES functions and hence scenario simulations can be directly linked to changes in the components of SES.

Using the proposed LV model, we analyze the following scenarios.

- *Business-as-usual* (*BAU*) current trends (in terms of population, economy, political/legislative and culture) are expected to continue.
- *Non-declining whites scenario* (*SW1*) Instead of declining, the SES shares of Whites in the forecasted period are stabilized around the value of the last historical observation. We calculate the increase in the SES function of Whites needed to ensure that their SES shares does not decline and we find that $0.04( t-T_{0})$, where $T_{0}$ represents the year 2013, provides this result. In other words, as it will be discussed in Sect. 4.2, the function $0.04( t-T_{0})$ allows to derive a measure of the magnitude of the changes that are necessary to achieve a given result (in this case stabilizing the SES share of Whites at the value prevailing in 2013). We therefore modify the SES function of the Whites as follows
  9 $$\begin{aligned} f_{3}^{SW1}(t) =f_{3}(t) +0.04\left( t-T_{0}\right) ,\quad \forall t\in \left[ T_{0},T\right] \end{aligned}$$
  where $T_{0}$ and *T* represent the years 2013 and 2018, respectively.
- *Improving blacks’ SES scenario* (*SB1*) the SES shares of Blacks in the forecasted period increase at a faster rate than in the BAU, and becomes higher than that of Asians after the year 2016. We calculate by how much the SES function of the Blacks should be increased to obtain this result. We find that the function $0.07\left( t-T_{0}\right)$ ensures that the SES share of the Blacks becomes higher than that of the Asians after the year 2016.^3^ Given this result, we modify the SES function of Blacks as follows
  10 $$\begin{aligned} f_{2}^{SB1}(t) =f_{2}(t) +0.07\left( t-T_{0}\right) ,\quad \forall t\in \left[ T_{0},T\right] . \end{aligned}$$
- *Economic boom scenario* (*SEB*) In the forecasted period, the income and the employment rate of all the groups improve. In particular, we assume that the changes mirror the losses produced by the economic crisis. For instance, Blacks during the 2007-2010 economic crisis lost $1.82\%$ of their income, hence we simulate that during the forecasted period the income of the Blacks grows at most by $1.82\%$ *more than in the BAU* (by 2018). Then, we consider the following SES function
  11 $$\begin{aligned} f_{1}^{SEB}(t)= & {} f_{1}(t) +\epsilon _{1}\left( \dfrac{ t-T_{0}}{T-T_{0}}\right) ^{2},\nonumber \\ f_{2}^{SEB}(t)= & {} f_{2}(t) +\epsilon _{2}\left( \dfrac{ t-T_{0}}{T-T_{0}}\right) ^{2} ,\quad \forall t\in \left[ T_{0},T\right] , \nonumber \\ f_{3}^{SEB}(t)= & {} f_{3}(t) +\epsilon _{3}\left( \dfrac{ t-T_{0}}{T-T_{0}}\right) ^{2}, \end{aligned}$$
  where $$\epsilon _{1}=-0.0909, \epsilon _{2}=-0.0577, \epsilon _{3}=-0.1178$$.

To highlight how to modify the main variables to obtain the SES functions in Eqs.  (9) and (10), we proceed as follows. Limiting our attention to Eq. (9), we assume that the changes in the SES function $f_{3}$ are due to the average income $I_{3}$ (*or* employment rates) only. This is because, in normal circumstances, life expectancy and group size are relatively inelastic in the short term. From Eqs. (4) and (6) we determine

12 $$\begin{aligned} f_{3}^{SW1}(t) =f_{3}(t) +\ln \dfrac{I_{3} ^{SW1}(t) }{I_{3}(t) },\quad \forall t\in \left[ T_{0},T\right] . \end{aligned}$$

Then, by comparing Eqs. (9) and (12), we obtain

13 $$\begin{aligned} I_{3}^{SW1}(t) =I_{3}(t) \exp \left( 0.04(t-T_{0})\right) ,\quad \forall t\in \left[ T_{0},T\right] . \end{aligned}$$

Analogously, if we assume that the change in the SES function $f_{3}$ is due to both the average income $I_{3}$ and employment $E_{3}$, instead of Eq. (13) we obtain

14 $$\begin{aligned} \alpha (t) \beta (t) =\exp \left( 0.04(t-T_{0} )\right) ,\quad \forall t\in \left[ T_{0},T\right] \end{aligned}$$

where $$\alpha (t) =E_{3}^{SW1}(t) /E_{3}(t) ,\beta (t) =I_{3}^{SW1}(t) /I_{3}(t)$$.

Alternatively, owing to Eq. (4), it is possible to study how future dynamics change on varying the driving variables $L_{i}, E_{i}, I_{i},N_{i}$ ($i=0,...,3$). And indeed, any change in these variables result in a modification of the SES functions and of the competitive roles as shown in Eqs. (6) and (8), respectively. In particular, we show how to modify the income and the employment rate of the four racial groups to obtain the SES functions in Eq. (11). From Eqs. (4) to (6), if we assume that the change in the SES functions is only due to the functions $E_{i}$ and $I_{i}$, for $i=1,2,3$, we can write

15 $$\begin{aligned} f_{i}^{SEB}(t) -f_{i}(t) =\ln \left[ \dfrac{ E_{i}^{SEB}(t) }{E_{i}(t) }\times \dfrac{ I_{i}^{SEB}(t) }{I_{i}(t) }\times \dfrac{E_{0}(t) }{E_{0}^{SEB}(t) }\times \dfrac{I_{0}(t) }{ I_{0}^{SEB}(t) }\right] ,\quad \forall t\in \left[ T_{0},T\right] \end{aligned}$$

where $E_{i}(t)$ and $I_{i}(t)$ refer to the BAU scenario. Setting

$$\begin{aligned} \dfrac{E_{i}^{SEB}(t) }{E_{i}(t) }=\alpha _{E_{i}}(t) ,\quad \dfrac{I_{i}^{SEB}(t) }{ I_{i}(t) }=\alpha _{I_{i}}(t) ,\quad i=0,...,3 \end{aligned}$$

Equation (15) becomes

16 $$\begin{aligned} f_{i}^{SEB}(t) =f_{i}(t) +\ln \left[ \dfrac{\alpha _{E_{i}}(t) \times \alpha _{I_{i}}(t) }{\alpha _{E_{0}}(t) \times \alpha _{I_{0}}(t) }\right] ,\quad \forall t\in \left[ T_{0},T\right] . \end{aligned}$$

To determine the analytical form of the functions

17 $$\begin{aligned} \dfrac{\alpha _{E_{i}}(t) \times \alpha _{I_{i}}(t) }{ \alpha _{E_{0}}(t) \times \alpha _{I_{0}}(t) },\quad i=1,2,3 \end{aligned}$$

we proceed as follows. It is assumed a period of economic growth characterized by a “specular trend” of the economic crisis of the years 2007–2010. Then, owing to the historical data of the income and the employment rate of the four groups, we have

18 $$\begin{aligned} \alpha _{E_{0}}\left( T\right)&=1.0762,\quad \alpha _{I_{0}}\left( T\right) =1.0865, \nonumber \\ \alpha _{E_{1}}\left( T\right)&=1.0444,\quad \alpha _{I_{1}}\left( T\right) =1.0223, \nonumber \\ \alpha _{E_{2}}\left( T\right)&=1.0840,\quad \alpha _{I_{2}}\left( T\right) =1.0182, \nonumber \\ \alpha _{E_{3}}\left( T\right)&=1.0480,\quad \alpha _{I_{3}}\left( T\right) =0.9917. \end{aligned}$$

In addition, if the effect of economic growth is supposed to result in a gradual change in SES functions, we can model these functions by second-order polynomials. Then, since $\alpha _{E_{i}}\left( T_{0}\right) =\alpha _{I_{i}}\left( T_{0}\right) =1, i=0,...,3$, we obtain

19 $$\begin{aligned} \ln \left[ \dfrac{\alpha _{E_{i}}(t) \times \alpha _{I_{i}}(t) }{\alpha _{E_{0}}(t) \times \alpha _{I_{0}}(t) }\right] =\dfrac{\alpha _{E_{i}}\left( T\right) \times \alpha _{I_{i}}\left( T\right) }{\alpha _{E_{0}}\left( T\right) \times \alpha _{I_{0}}\left( T\right) }\left( \dfrac{t-T_{0}}{T-T_{0}} \right) ^{2},\quad \forall t\in \left[ T_{0},T\right] . \end{aligned}$$

Hence, Eq. (16) becomes

$$\begin{aligned} f_{i}^{SEB}(t) =f_{i}(t) +\epsilon _{i}\left( \dfrac{ t-T_{0}}{T-T_{0}}\right) ^{2},\quad \forall t\in \left[ T_{0},T\right] \end{aligned}$$

where

$$\begin{aligned} \epsilon _{i}=\dfrac{\alpha _{E_{i}}\left( T\right) \times \alpha _{I_{i}}\left( T\right) }{\alpha _{E_{0}}\left( T\right) \times \alpha _{I_{0}}\left( T\right) },\quad i=1,2,3. \end{aligned}$$

Finally, from Eq. (18) we have

$$\begin{aligned} \epsilon _{1}=-0.0909,\quad \epsilon _{2}=-0.0577,\quad \epsilon _{3}=-0.1178. \end{aligned}$$

The scenarios that we analyze are reported in Table 5.

**Table 5:** Different scenarios captured by suitable SES functions in the period 2013–2018

Scenarios	SES functions		
	Asians	Blacks	Whites
BAU	${f}_{1}(t)$	${f}_{2}(t)$	${f}_{3}(t)$
SW1	${f}_{1}(t)$	${f}_{2}(t)$	${f}_{3}(t) +0.04(t-T_{0})$
SB1	${f}_{1}(t)$	${f}_{2}(t) +0.07(t-T_{0})$	${f}_{3}(t)$
SEB	$f_{1}(t) -0.0909\left( \dfrac{ t-T_{0}}{T-T_{0}}\right) ^{2}$	$f_{2}(t) -0.0577\left( \dfrac{ t-T_{0}}{T-T_{0}}\right) ^{2}$	$f_{3}(t) -0.1178\left( \dfrac{ t-T_{0}}{T-T_{0}}\right) ^{2}$

## Results and discussion

In this section, we present and discuss the historical data and the results of the quantitative analysis performed by means of the proposed LV model. All the forecasting in the scenario analysis extends to the year 2018 (see Table 5). As a preliminary remark, we note that the interactions most frequently observed are mutualism and competition. The former implies that an increment in the SES of one group has a positive effect on the SES of the other group, whereas the latter means that an increment in the SES of one group has a negative effect on the SES of the other. A more detailed description of the competitive roles can be found in the Sect. Material and Methods.

### Historical data, and BAU scenario

**Fig. 6:**
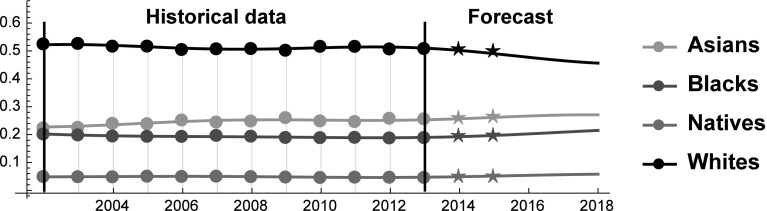
SES Shares of Asians, Blacks, Natives and Whites over the period 2002–2018. Star symbols represent the observed SES shares in the years 2014 and 2015

Before describing the results, we can make some preliminary remarks. First, the SES of Whites is substantially higher than the SES of the other racial groups, indicating that they stand in a position of relative dominance. Yet, their dominance is closely related to their numerosity, because Asians have higher values than Whites in all the other indicators (see Fig. 1). Because the relative number of Whites is expected to decline, their relative dominance is likely to weaken. Moreover, Asians are eroding SES shares from all the other groups. This result is mainly driven by their larger relative improvements in terms of income and employment rates. On the contrary, Blacks have the lowest growth rate in terms of income, while their employment level is lower in 2012 than in 2002. The bad performances of Blacks in these dimensions are only partially compensated by their improvements in expected life. As a result, between 2002 and 2013 the gap between Asians and Blacks increased. Interestingly, in the forecasted future these patterns are expected to partially change (Fig. 6).

**Fig. 7:**
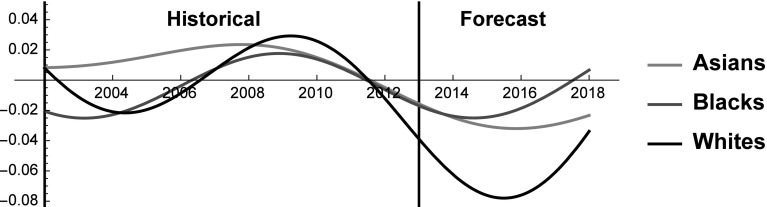
Interaction coefficients of Asians, Blacks and Whites 2002–2018

An interesting explanation is that Blacks—being the weakest group – thrive when there is mutualism in the society, whereas they suffer when society is characterized by rivalrous interactions (see Fig. 7). And indeed, before the crisis the rate of growth of the SES share of the Black population had a positive sign only between 2006 and 2007. Incidentally, these years are preceded by mutualism between Whites and Blacks (see Fig. 7). As the model forecasts that groups proceed in mutualism between 2013 and 2017, this might explain why Blacks perform well in the forecasted period.

Let us now turn to analyzing the kind and the intensity of the interactions among Asians, Blacks and Whites by studying the behavior of the interaction coefficients (see Fig. 7). Notably, the model captures the simultaneous interaction of all the groups, which is an important advantage of Lotka–Volterra models.

The most relevant finding is that the groups enter into pure competition roughly at the beginning of the crisis, and the interaction coefficients of all the three groups reach their maximum in 2009, during the *spannung* of the crisis. At that moment, the groups invert the tendency of the previous years (i.e. the derivatives of the interaction coefficient become negative) and steadily decrease the intensity of the competition to reach mutualism in 2012. A possible explanation is that negative economic conditions induce groups to compete to appropriate the scarce resources available. Yet, when the intensity of the crisis reaches a certain threshold the fear of potential losses induces the groups to slowly abandon competitive behaviors in favor of more mutualistic interactions. We remark that in 2009 the GDP of the United States decreased by 2.9%, whereas in 2008 it only decreased by 0.3%. Therefore, the losses to be allocated among the groups in 2009 were significantly larger than in the preceding years. This behavior during severe crisis is in line with the well established concept of loss aversion. That is, individuals fear losses more than they value gains (Tversky and Kahneman 1991), and hence during severe crisis the groups might avoid win-lose interactions for fear of being on the losing side. Let us now move to analyze the single dyads of interaction. During the period 2002–2013 Asians and Blacks always engage in rivalrous interactions (prey-predator between 2002 and 2006, and pure competition between 2006 and 2013), thus disproving the idea that minorities cooperate, wittingly or unwittingly, to erode the privileges of the dominant majority. A possible explanation is that the two groups present drastically different traits, as Asians are only rarely unemployed, have significantly higher incomes and have a higher life expectancy than Blacks. Therefore, it is very likely that in the political arena the two groups take diametrically opposite positions on many divisive issues. Two additional points should be made. A similar pattern is observed also between Asians and Whites, as the groups always engage in rivalrous interactions during the period 2003–2013. This finding is in line with the well known group position theory, which predicts that the dominant group reacts in an aggressive manner when exposed to the growing power of a minority that threatens the prevailing hierarchical structure of the society (Blumer 1958). Last, we turn to Blacks/Whites interaction. Here, we observe the only instance of mutualism in the years preceding the crisis. This mutualistic relationship could be interpreted as an attempt to cooperate to prevent Asians from eroding other groups SES shares. This attempt abruptly ended when the resources available became scarcer during the crisis.

### Non-declining whites scenario (SW1)

**Fig. 8:**
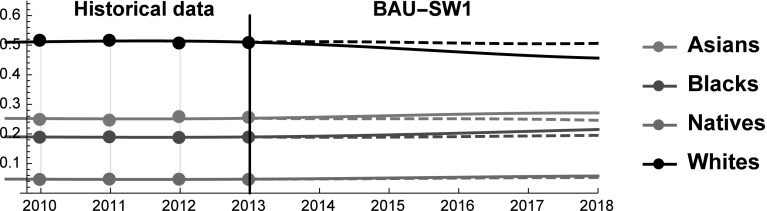
Scenario SW1. SES shares of Asians, Blacks, Natives, and Whites. Continuous and dashed lines refer to BAU and SW1 scenarios, respectively

In the BAU, the behavior of SES shares reveals that the dominant position of Whites is being eroded by other ethnic groups, and especially by Asians. The only factor that keeps Whites’ SES above that of Asians is the relative numerosity of the two groups. As United States is rapidly turning into a majority–minority society, the relative size of the White population *vis-à-vis* the other groups is going to shrink thus accelerating the decline of their SES. History teaches that when the dominant group feels threatened it can adopt defensive behaviors and/or support nationalistic parties (e.g. the 2016 Austrian presidential elections). According to many, the United States are already experiencing a white backlash (“whitelash”) and the election of President Donald Trump would be a proof of this (Gusterson 2017). The practical relevance of the Whitelash is testified by the fact that it is discussed in scientific articles [e.g. (Gusterson 2017)], in popular media (Ryan 2016) and in university courses (e. g. “White Backlash in a Dramatically Changing Landscape” (Prof. Roithmayr), Yale Law School).^4^ Also the tax-cut recently proposed by President Donald Trump can be seen as a signal that the US might be moving in this direction. And indeed, according to some the proposed tax-cut provides relatively small benefits to the middle class, while rewarding more the richest (Nitti 2017). Although at the moment of writing any comment on the possible tax reform amounts only to speculation, a tax reform that benefits the wealthiest would indirectly harm the weaker racial groups, and therefore it would contribute to protect the status relatively wealthier groups. For this reason, in this scenario we study what kind of policies are sufficient to keep Whites’ SES constant in the forecasted period 2013–2018. In particular, as life expectancy and group size are inelastic in the short term, we study what changes in employment rates and income level are necessary to prevent Whites from losing SES shares in the forecasted future (see Fig. 8).

We find that to prevent Whites’ SES from declining their income or occupational level must be increased to reach a + 22% with respect to the BAU in 2018 [see Eq. (13)]. Acting on employment alone is not a viable strategy, because—for obvious reasons—it cannot be increased by + 22% (see Fig. 1). Therefore, the only two available options are to increase income alone or to simultaneously act on employment and income according to Eq. (14). In any case, even when acting simultaneously on both variables the required changes remain very large (still above the + 10.5% threshold in 2018).

**Fig. 9:**
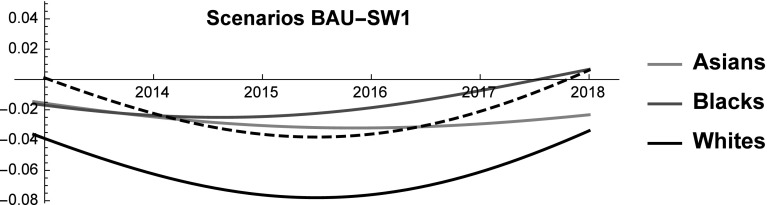
Scenario SW1. Competitive roles of Asians, Blacks and Whites. Continuous and dashed black lines refer to BAU and SW1 scenarios, respectively

Net of any ethical consideration on the desirability of this goal, the analysis reveals that it is almost impossible to prevent Whites from losing SES shares by implementing policies that act on income and employment rate in the short-medium term. If a political party decides to embark on a crusade to preserve the dominant position of Whites, it must be willing to either work on long-term policies or to take rather extreme measures. Interestingly, we find that in this scenario Whites reduce their level of mutualism toward the other groups and by 2018 they engage in rivalrous interactions with both Asians and Blacks (see Fig. 9). This result is probably dictated by the new-found strength of Whites that no longer feel the need of cooperating with other ethnic groups.

### Improving the SES of blacks (SB1)

**Fig. 10:**
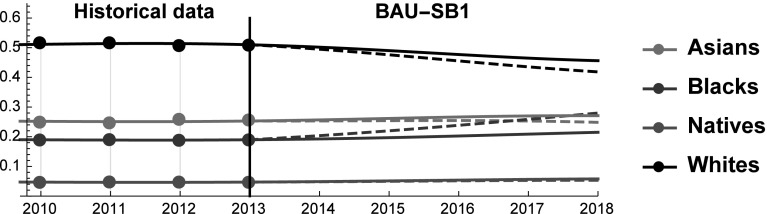
Scenario SB1. SES shares of Asians, Blacks, Natives, and Whites. Continuous and dashed lines refer to BAU and SB1 scenarios, respectively

Blacks are the weakest group of the three considered. This finding is particularly striking if we consider that their number is significantly higher than that of Asians. Therefore, it is possible that in the near future there will be (more) political pressure toward an increased equality among racial groups. The surge of the “Black Lives Matters” movement originated in Ferguson, MO after a police officer killed Michael Brown is a clear symptom of this pressure. The fact that president Barack Obama set up a dedicated task force testifies that the voice of the “Black Lives Matters” movement has been heard at the highest political level and might affect future policies (Shannon 2017). Moreover, the recent increase in Congress representatives of colour may ease the implementation of pro-equality policies (Manning 2017). In this vein, in this scenario we test by how much the income and/or the employment rate of the Black population should be improved to ensure that their SES reaches that of the Asians (see Fig. 10). We remark that Blacks are 2.5 times more numerous than Asians and therefore an equal SES implies that Asians still perform better on the job market and in terms of life expectancy.

**Fig. 11:**
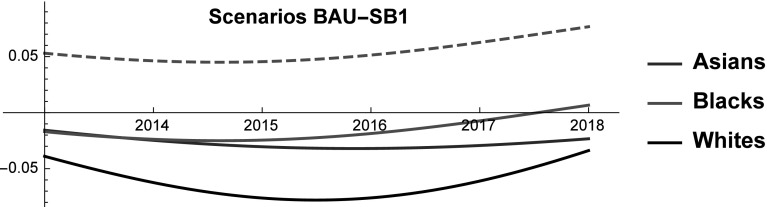
Scenario SB1. Competitive roles of Asians, Blacks, and Whites. Continuous and dashed red lines refer to BAU and SB1 scenarios, respectively

Ensuring that the SES of Blacks is equal to that of Asians requires increasing their income to reach a + 42% with respect to the BAU. As above, a possible alternative is to simultaneously act on income and employment according to Eq. (14) (about + 19% in 2018). The required changes remain implausible (see Fig. 1). There are a number of ways to improve employment rate or the average income of Blacks. Among them, the most notable are enhancing affirmative action policies, targeting discriminatory practices, increasing tax rates for high income groups while decreasing tax rates for low income groups, etc. However, these policies generally produce effects that are significantly smaller than the one required to ensure that Blacks and Asians have the same SES. For example, there is an extensive literature trying to identify which share of the wage gap between Blacks and Whites can be explained by racial discrimination (Fryer et al. 2013). The findings of this strand of literature are conflicting as some authors argue that wage differential is mainly driven by a gap in skills between Blacks and Whites (Neal and Johnson 1996), whereas others find that racial discrimination explains 60% of the wage differential (Reimers 1983). A recent article locates in between these extremes and finds that racial discrimination accounts for about 30% of the wage gap (Fryer et al. 2013). Assuming that 30% is a reasonable estimate, even eliminating all racial discrimination in wage determination would not suffice to allow Blacks to enjoy the same SES as Asians. And indeed, the difference between the income of Blacks and that of Whites in 2013 is equal to 11.150$. Therefore, eliminating the part of the gap that is due to discrimination can only increase the income of Blacks at best by around 12% (or 3.345$). It is unlikely that reducing the gap between the income of Blacks and that of Whites can be achieved by simply increasing the income of the former. Even though it is not necessarily a zero-sum-game, it is reasonable to assume that the values would converge, with Whites average income decreasing and Black average income increasing. Similarly, affirmative action policies have been shown to be effective in improving minorities conditions (Miller and Segal 2012), yet the size of the effect that they generate is significantly smaller than what is required by this scenario. These findings suggest that to promote equality among racial groups it is mandatory to act on long term variables like education.

Last, in this scenario Blacks abandon any mutualism in favor of rivalrous interaction with both Asians and Whites (see Fig. 11). It seems that when the gap between the disadvantaged and the stronger groups decreases below a given threshold the former engages in rivalrous interaction to further improve its condition in society and reach the status of other groups. A corollary of this hypothesis would be that to reduce differences among racial groups it is necessary go through periods of social frictions.

### Economic boom (SEB)

Last, we study how a period of economic growth affects the interaction among racial groups and the dynamics of their SES shares. Differently from the first two scenarios we do not set a target (i.e. keeping the SES share of the Whites constant or increasing the SES share of the Blacks to the level of that of Asians). Instead, we study the impact of a change in two SES variables on SES dynamics. Therefore, from a mathematical perspective this scenario is the other side of the coin of the first two. As we intend to portray a period of economic expansion, we act on the income and the employement rate of the four groups. The reason is twofold. On the one hand, these components of the SES are conceptually more immediately related to changes in macroeconomic trends. On the other hand, life expectancy and group size are less elastic in the short term. Clearly, we have no way to predict the magnitude of an economic boom or how the benefits would be allocated between racial groups with precision. For this reason, we simulate a scenario in which SES dynamics mirror those of the economic crisis. For instance, Asians during the 2007–2010 economic crisis lost $2.22\%$ of their income, hence we simulate that during the forecasted period the income of the Asians grows at most by $2.22\%$ *more than in the BAU* (by 2018). Besides offering a slightly less arbitrary anchor, this choice has an interesting property. As Blacks and Natives are the groups the suffered more the crisis, in the simulation performed these two groups enjoy larger *additional* gains from the economic expansions. In principle, in absolute terms the Natives and the Blacks might still grow *less* than Asians and Whites, because we add these value on the base trends predicted by the BAU. In other words, if the base growth of Whites’ income is significantly higher than that of Blacks, the overall increase in income of the Whites might still be larger than that of the Blacks. Thus, we do not postulate that during an economic boom the Blacks and the Natives will necessarily perform better than the Asians and the Whites. Instead, in line with the economic literature, we claim that an economic expansion can contribute to lessening the racial gap and benefit weaker groups (compared to the performance of each group in BAU), both in terms of unemployment rate (Couch and Fairlie 2010) and in terms of income (Freeman and Rodgers 2000; Wilson and Rodgers 2016).

**Fig. 12:**
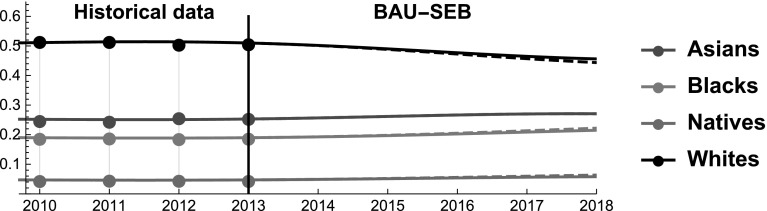
Scenario SEB. SES shares of Asians, Blacks, Natives, and Whites. Continuous and dashed lines refer to BAU and SEB scenarios, respectively

**Fig. 13:**
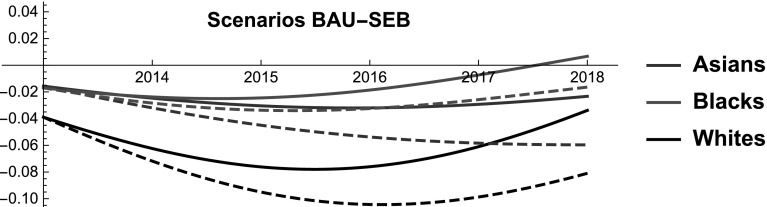
Scenario SEB. Competitive roles of Asians, Blacks, and Whites. Continuous and dashed red lines refer to BAU and SEB scenarios, respectively

**Fig. 14:**
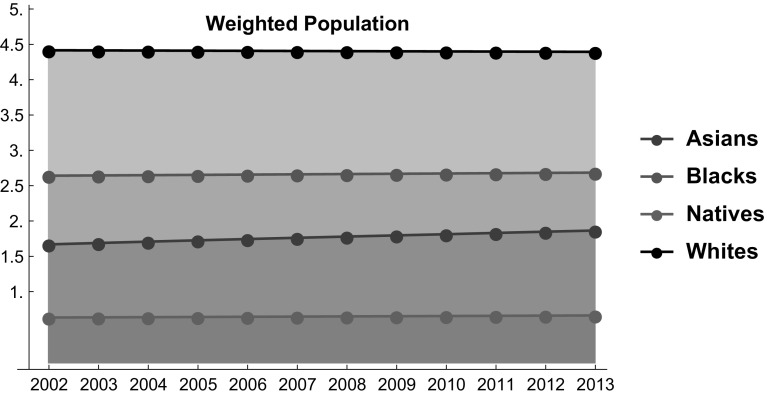
Weighted population size (e.g. $\ln \left( 1+{100}N_{i}/N\right)$, $i=0,\ldots ,3$) over the period 2002–2013

At a general level, we note that the simulated economic expansion has a negligible impact on SES shares (see Fig. 12), whereas it alters the interaction coefficients in a noticeable way (see Fig. 13). This finding is consistent with the first two scenario simulations. It confirms that altering SES dynamics in the short-medium term is extremely hard, whereas the kind of inter-group interaction is more malleable.

More in detail, we notice that during the simulated economic boom the SES shares of all the groups remain almost identical. The minimal changes observed point to a very small convergence of the SES shares, given that Blacks and Natives perform marginally better than in the BAU, while the Whites perform marginally worse. The finding that—albeit only marginally—an economic expansion can lead to lessening the gap between the strongest and the weakest groups is consistent with the results obtained by the economic literature (Wilson and Rodgers 2016). At the same time, we observe that the interaction coefficients of the groups significantly change in the simulated scenario. The mutualism among racial groups becomes much stronger than in the BAU and the rivalrous interactions disappear completely. In other words, as the size of the pie increases the groups shift to more symbiotic relationships. This seems to be in line with the observation that during the economic crisis all the groups engaged in fierce competition.

## Conclusion and research perspectives

Racial groups interactions are an important component of the dynamics underlying the functioning of many modern societies. Yet, the growing racial diversity within many countries is making their study—especially from a quantitative perspective—increasingly complex. For example, dyadic studies analyzing Blacks/Whites relations are not apt to capture the dynamics characterizing the modern American society. In this work, after defining a SES index for each racial group and rewriting each of them as a logit model, we employ an integrable nonautonomous LV model to analyze quantitatively and from a dynamic perspective the kind and the intensity of the interaction among these groups. This approach has a number of advantages. First, it can capture nuanced forms of interaction like mutualism and commensalism. Second, as the interaction coefficients are explicitly dependent on time, it allows to study how the interaction evolves over time. Third, because the solutions of the LV systems are known, the analysis is not data demanding and the interaction coefficients of the model do not have to be estimated via expensive numerical methods. Last, when combined with the scenario method, this model helps to understand and shape the evolution of social dynamics. We apply the model to the study of the interactions in terms of SES among Asians, Blacks, Native Americans and Whites in the United States. However, we remark that our model is general and therefore it is possible to include more groups (e.g. Hispanics) or to focus on different continuous measures of SES. In particular, we build an index of SES to study how racial group interactions evolve over time. To build this index we aggregate data on life expectancy, average income, employment rate and group numerosity for each group during the period 2002–2013. Our analysis highlights that racial interactions are inherently dynamic and influenced by macroeconomic factors. Moreover, in a scenario framework we study how the SES of the groups should be altered to achieve the following goals: (i) preventing the SES share of Whites from declining and (ii) ensuring that the SES share of Blacks reaches that of Asians. The main finding is that these goals require changes in groups’ SES that are hard to achieve via short-term policies. Last (iii), we investigate the impact of an economic boom on interracial dynamics. We find that the SES shares are hardly affected by an economic expansions, and that mutualistic inter-group interactions prevail over rivalrous interactions when the state of the economy is improving.

### Electronic supplementary material

Below is the link to the electronic supplementary material.

Supplementary material 1 (xlsx 14 KB)

## Appendix

See Table 6 and Fig. 14.

**Table 6:** SES shares over the period 2002–2013

Time	SES index			
	Asians	Blacks	Natives	Whites
2002	0.223783	0.201804	0.0493768	0.525036
2003	0.225786	0.198635	0.0488564	0.526722
2004	0.240203	0.194604	0.0483301	0.516863
2005	0.238631	0.193598	0.0506537	0.517117
2006	0.252068	0.193361	0.0497493	0.504821
2007	0.244759	0.196983	0.0508383	0.507421
2008	0.248494	0.1941	0.0490648	0.508341
2009	0.260717	0.189532	0.0475419	0.50221
2010	0.248889	0.18878	0.0460497	0.516281
2011	0.246458	0.189982	0.0472955	0.516265
2012	0.258396	0.187785	0.0469006	0.506919
2013	0.256209	0.189128	0.0462525	0.50841

## Footnotes

1 The Census does not include the data relative to the income of Native Americans in the same table. Therefore, we derived it from the data on FactFinder of the Census Bureau, https://factfinder.census.gov/faces/nav/jsf/pages/index.xhtml.

2 The data on 2014 and 2015 was not available at the time in which the article was written. We included this data at a later stage to compare the predictions of our model with the observed dynamics.

3 As for Non-declining Whites scenario, Sect. 4.2 further elaborates on this point.

4 Description of the course available at: http://courses.law.yale.edu/courses/term/18.
